# Variations in *LOXL1* associated with exfoliation glaucoma do not affect amine oxidase activity

**Published:** 2012-01-31

**Authors:** Seonkwan Kim, Youngho Kim

**Affiliations:** Department of Biochemistry, School of Medicine, Wonkwang University, Sinyong-Dong 344-2, Iksan-City, Jeollabuk-Do 570-749, South Korea

## Abstract

**Purpose:**

Lysyl oxidase-like 1 (LOXL1) is a copper-dependant amine oxidase that plays an essential role in elastogenesis. Two non-synonymous single-nucleotide polymorphisms of *LOXL1*, R141L (rs1048661) and G153D (rs3825942), have been reported to significantly increase susceptibility to exfoliation glaucoma (XFG). To evaluate the impact of the R141L and G153D variations on the amine oxidase activity of LOXL1, we generated four different haplotypes of LOXL1 with R141L and G153D and assessed the amine oxidase activity of the LOXL1 variant proteins.

**Methods:**

The four different haplotype variants of LOXL1 were created by oligonucleotide-directed mutagenesis in an LOXL1 expression vector. Recombinant LOXL1 variant proteins were purified by nickel-affinity chromatography. The amine oxidase activities of the LOXL1 variant proteins were assessed using peroxidase-coupled fluorometric assays.

**Results:**

All of the haplotype variants of LOXL1 (141R-153G, 141R-153D, 141L-153G, and 141L-153D) showed β-aminopropionitrile-inhibitable amine oxidase activity toward elastin, type I collagen, and cadaverine, indicating that each LOXL1 variant functions as an amine oxidase. However, there were no significant differences in amine oxidase activity between the LOXL1 haplotype variants toward the tested substrates.

**Conclusions:**

The R141L and G153D variations in the NH_2_-terminal region of LOXL1 do not affect the amine oxidase activity of LOXL1. This is consistent with recent genetic findings on the reversal of risk alleles of R141L and G153D in different ethnic backgrounds. Our results suggest that other unknown genetic factors or molecular mechanisms may be more relevant to the development of XFG.

## Introduction

Lysyl oxidase-like 1 (*LOXL1*) was originally reported as a novel human gene with amino acid sequence homology to the COOH-terminus of lysyl oxidase (LOX) [1,2]. LOX is a copper-dependent amine oxidase responsible for the lysine-derived cross-links in collagen and elastin, modulating the essential step for the biogenesis of fibrillar extracellular matrix in most tissues [3]. The *LOX* gene family consists of five paralogs (*LOX*, *LOXL1*, *LOXL2*, *LOXL3*, and *LOXL4*) in humans, each containing a copper-binding motif, lysyl-tyrosyl-quinone (LTQ) residues, and a cytokine receptor-like (CRL) domain in the highly conserved COOH-terminus. We have previously shown that each of the LOX-like proteins functions as an amine oxidase [4-7], but functional differences of these LOX family proteins have not been clearly determined. Recently, *Loxl1* null mice were found to have significantly reduced levels of desmosine in various tissues, indicating that LOXL1 serves as the primary cross-linking enzyme for formation, maintenance, and remodeling of elastic fibers [8].

Exfoliation glaucoma (XFG) occurs in the context of the exfoliation syndrome (XFS), characterized by progressive accumulation of abnormal microfibrillar material throughout the anterior segment of the eye and various ocular tissues [9]. XFG is the most common identifiable cause of secondary open-angle glaucoma worldwide, causing rapid progression of glaucomatous optic neuropathy with a high resistance to medical treatments [9]. The abnormal microfibrillar deposits in XFG are composed of a crosslinked and insoluble glycoprotein-proteoglycan complex, predominantly with epitopes of elastic fibers [10], suggesting the relevance of LOXL1 to the formation of the exfoliative material. In fact, using direct mass spectrometry, LOXL1 was identified as a component of the exfoliative material surgically isolated from XFG patients [11]. Later, using atomic force microscopy-based imaging, LOX1 was found to be localized around the fibrous protein material on the lens capsule surface [12].

In a genome-wide search for genetic risk factors of XFG, two non-synonymous single-nucleotide polymorphisms (SNPs) in exon 1 of *LOXL1*, rs1048661G/T (R141L) and rs3825942G/A (G153D), were identified with the G allele as a risk allele at both SNPs in Nordic populations [13]. Of four possible haplotypes involving the two SNPs, only three haplotypes (GG, GA, and TG) were detected, and the TA haplotype was not observed in either the XFG or control group. Relative to the GA haplotype, the GG and TG haplotypes showed odds ratios of 27.05 and 8.90, respectively, for the incidence of XFG [13]. Subsequent studies confirmed the association of these SNPs with XFG in different populations, including German, Italian, Caucasian Australian, and United States populations [14-17].

To evaluate the effects of the R141L and G153D variations on the amine oxidase activity of LOXL1, we constructed the GG, GA, TG, and TA haplotype variants across rs1048661 and rs3825942 by oligonucleotide-directed mutagenesis, each corresponding to 141R-153G, 141R-153D, 141L-153G, and 141L-153D in the amino acid sequence of LOXL1, respectively. The LOXL1 variant proteins were expressed and purified as hexa-histidine tagged recombinant proteins. The amine oxidase activities of the purified LOXL1 variant proteins were accessed using in vitro peroxidase-coupled fluorometric assays. The four different haplotype variants of LOXL1 did not show significant differences in amine oxidase activity toward elastin, type I collagen, or cadaverine.

## Methods

### Construction of expression plasmids for four different *LOXL1* haplotypes

The expression plasmid for *LOXL1* was generated as previously described [4]. A pET21a-derived expression plasmid containing the nucleotide sequence from codon 135D to the COOH-terminus was used as a template for oligonucleotide-directed mutagenesis. The mutagenesis was performed using the QuikChange II site-directed mutagenesis kit (Stratagene, La Jolla, CA) according to the manufacturer’s protocol. Thermocycling consisted of 16 cycles at 95 °C for 30 s, 58 °C for 1 min, and 68 °C for 7 min. The sequences of the oligonucleotide primers used for mutagenesis are listed in Table 1. The nucleotide sequences of all resulting constructs were confirmed by DNA-sequencing analysis using the BigDye™ Terminator Cycle Sequencing Ready Reaction Kit (Applied Biosystems, Foster City, CA) according to the manufacturer’s protocol.

**Table 1:** List of primers used for oligonucleotide-directed mutagenesis.

**SNP**	**Amino acid change**	**Sequence**	**Tm (°C )**
rs1048661	R→L	F-5’- gggcatggcccTggcccgcacctcc -3’	76
		R-5’- ggaggtgcgggccAgggccatgccc -3’	76
rs3825942	G→D	F-5’- acggcacggggActccgcctcctcg -3’	75
		R-5’- cgaggaggcggagTccccgtgccgt -3’	75

In the sequence, the bases mismatched with the template are indicated in capital letters.

### Purification and refolding of the haplotype variant proteins of LOXL1

Purification and refolding of the four different haplotype proteins of *LOXL1* were performed as we previously described for the LOX family proteins [4,7]. Briefly, the pET21a-derived expression constructs harboring the four different haplotypes of LOXL1 were introduced into the *Escherichia coli* strain BL21(DE3; Novagen, Darmstadt, Germany). After induction with 1 mM isopropyl-1-thio-β-d-galactopyranoside (IPTG) at 37 °C, the transformed bacterial cells were lysed in a buffer containing 50 mM Tris, pH 8.0, 1 mM EDTA, 100 mM NaCl, 1 mM PMSF, 1 mg/ml lysozyme, 1% Triton X-100, and 0.1 mg/ml DNase. After centrifugation, inclusion bodies were homogenized in a buffer containing 6 M urea, 10 mM K_2_HPO_4_, pH 8.2, and 3 mM β-mercaptoethanol. The LOXL1 variant proteins were then purified using Ni-NTA agarose resins (Qiagen, Valencia, CA) according to the manufacturer’s protocol. For refolding of the LOXL1 variant proteins, stepwise dialysis was performed in a buffer containing 10 mM K_2_HPO_4_, pH 9.6, 200 μM CuCl_2,_ and 2% sodium *N*-lauroylsarcosinate and then in a buffer containing 10 mM K_2_HPO_4_, pH 9.6, and 5 μM CuCl_2_. The protein samples were further dialyzed in 10 mM K_2_HPO_4_, pH 9.6. The dialyzed proteins were lyophilized in the presence of 10 mM trehalose using a freeze dryer (Labconco, Kansas City, MO). All purification procedures were performed at 4 °C. The purity and sizes of the LOXL1 variant proteins were determined using sodium dodecyl sulfate-PAGE (SDS–PAGE).

### Amine oxidase assays for the haplotype variant proteins of LOXL1

Amine oxidase activity of the haplotype variants of LOXL1 was assessed using a peroxidase-coupled fluorometric assay with the Amplex red hydrogen peroxide assay kit (Molecular Probes, Eugene, OR), as previously described [18]. Each reaction contained 10 μg of a purified LOXL1 variant protein and 20 pmol of substrate in a reaction volume of 200 μl. Parallel assays were performed in the absence or presence of 1 mM β-aminopropionitrile (BAPN) for 2 h at 37 °C. Bovine neck ligament elastin, calfskin type I collagen, and cadaverine (1,5-pentanediamine; Sigma-Aldrich, St. Louis, MO) were used as substrates for amine oxidase assays. Fluorescence was measured using a fluorescence microplate reader (BioTek, Winooski, VT) with excitation and emission wavelengths of 560 nm and 590 nm, respectively. Total amine oxidase activity was expressed as nM of H_2_O_2_ produced per μg of the LOXL1 variant protein, calculated by interpolation with fluorescence values from an H_2_O_2_ calibration curve.

## Results

**Figure 1:**
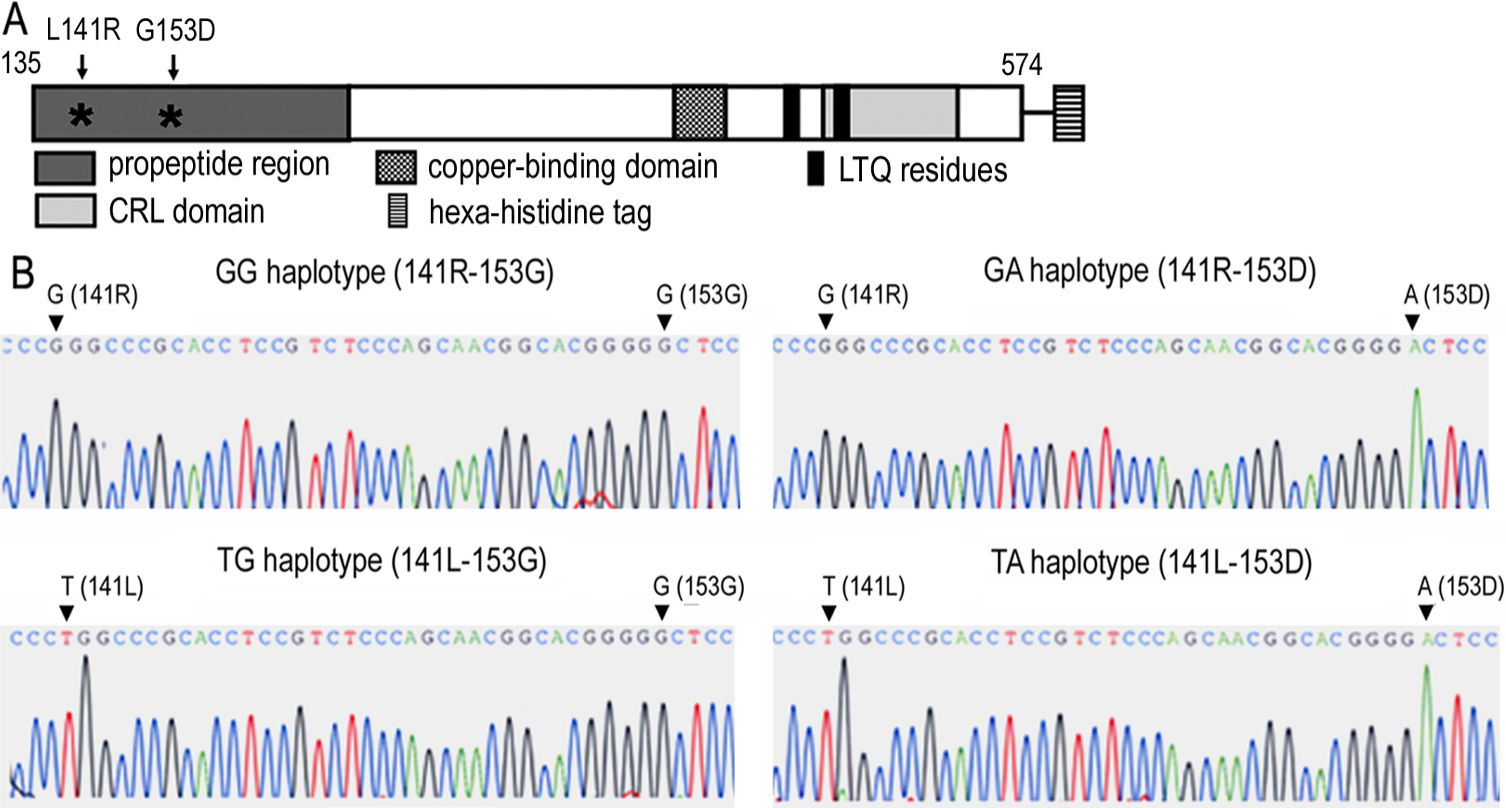
Construction of the *LOXL1* expression constructs for the four different haplotypes. **A**: A schematic diagram of the recombinant LOXL1 protein expected from the *LOXL1* expression constructs. The recombinant LOXL1 protein contains the amino acid sequence from codon 135D to the COOH-terminus with a hexa-histidine tag at the end. The approximate positions of R141L and G153D in the propeptide region are indicated with asterisks. **B**: DNA sequencing analysis of the mutated *LOXL1* expression constructs. The GG haplotype construct (141R-153G) was used as a template for oligonucleotide-directed mutagenesis. The amino acid sequence corresponding to each haplotype is indicated in parentheses. The nucleotide sequences at rs1048661 and rs3825942 are indicated with arrows.

We previously reported the expression and purification of an enzymatically active form of human LOXL1 as a recombinant protein (LOXL-p1) [4]. The NH_2_-terminal site of the previously reported LOXL-p1 [4] was deduced from amino acid sequence homology with the bovine LOXL1 protein, of which potential NH_2_-terminal proteolytic cleavage sites were suggested by incubation with bone morphogenetic protein (BMP)-1 [19]. Using the previously reported expression construct of LOXL-p1 (141R-153G haplotype) as a template, we generated three additional haplotype variants at R141L and G153D by a series of oligonucleotide-directed mutagenesis (Figure 1). All of the expected haplotype variants of LOXL1 (141R-153G, 141R-153D, 141L-153G, and 141L-153D) contain a copper-binding domain, a CRL-domain, and LTQ residues (477K and 511Y) in the COOH-terminal region, which are considered prerequisites for amine oxidase activity of the LOX family proteins.

**Figure 2:**
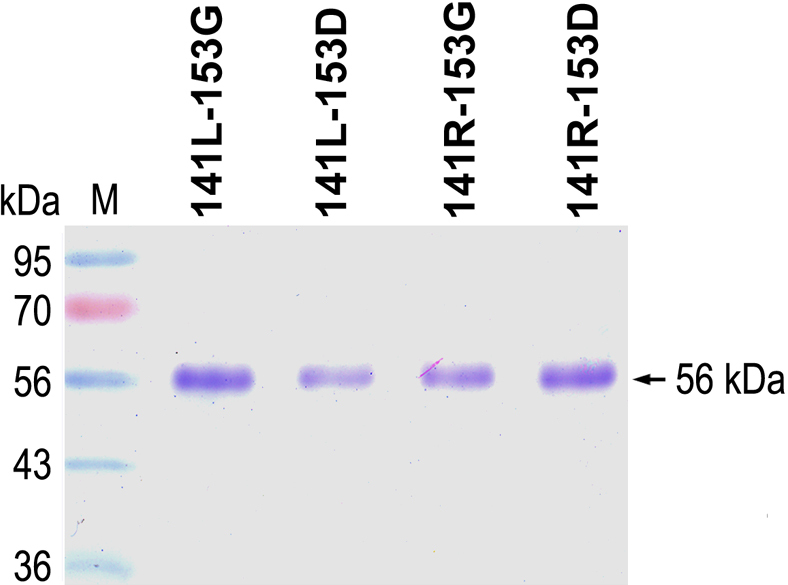
Expression and purification of the four different haplotype proteins of LOXL1. Each lane contains approximately 3 μg of purified recombinant LOXL1 protein of the 141L-153G, 141L-153D, 141R-153G, or 141R-153D haplotype. Lane M contains a molecular mass standard.

Upon induction with 1 mM IPTG in *E. coli*, all expression constructs of the four different LOXL1 variants showed high levels of expression. However, the LOXL1 variant proteins were within inclusion bodies. The insoluble fractions were solubilized with 6 M urea, and then the LOXL1 variant proteins were purified by nickel-chelating affinity chromatography using a hexa-histidine tag attached at the COOH-termini of the variant proteins. To refold the LOXL1 variant proteins denatured by urea during purification, the protein samples were subjected to stepwise dialysis in the presence of *N*-lauroylsarcosinate and Cu^2+^. The apparent sizes of the purified recombinant LOXL1 variant proteins were in good agreement with the deduced molecular mass, 56 kDa, and were over 95% pure on SDS–PAGE gels (Figure 2).

**Figure 3:**
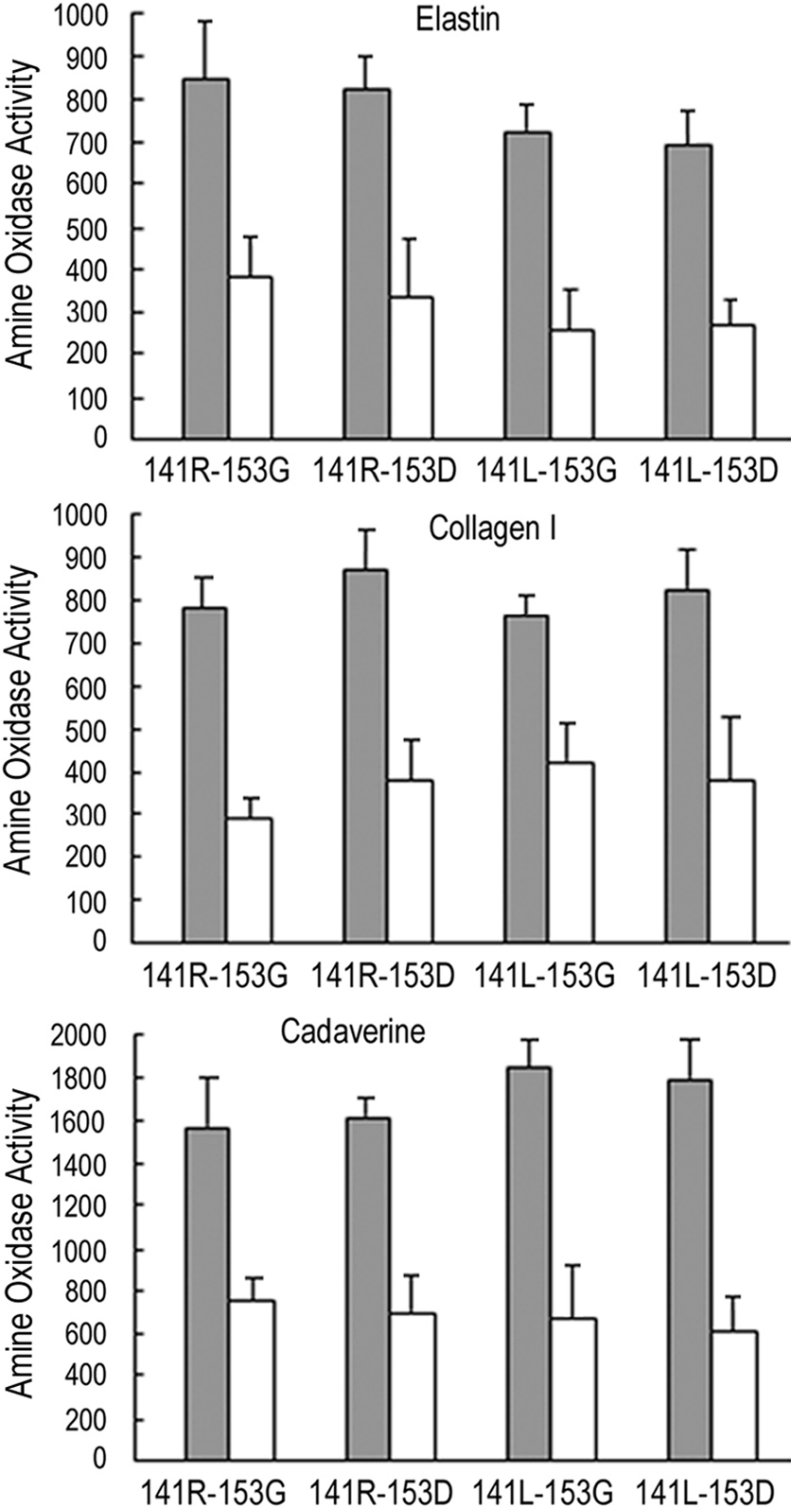
Amine oxidase activity of the four different haplotype variants of LOXL1 toward elastin, type I collagen, or cadaverine. The reactions without BAPN are in gray, and the reactions with BAPN are in white. The amine oxidase activity is expressed as nM of H_2_O_2_ produced per μg of the LOXL1 variant protein. Standard deviations are indicated in each graph, and the p-values between the reactions with and without BAPN are less than 0.05 in all cases tested.

Amine oxidase activity of the four different haplotype variants of LOXL1 was evaluated using physiologic substrates of LOXL1, elastin and type I collagen, as well as cadaverine, an artificial substrate with a diamine structure. All four haplotype variants of LOXL1 showed significantly higher levels of amine oxidase activity in the absence of BAPN than in the presence of BAPN, a well known specific inhibitor of LOX and LOXL1, indicating that the purified LOXL1 variant proteins had BAPN-inhibitable amine oxidase activity toward the tested substrates (Figure 3). Regardless of the haplotype, the LOXL1 variant proteins showed higher activities toward cadaverine than elastin and type I collagen. However, there were no significant differences in amine oxidase activity between the different haplotype variants toward any of the tested substrates (Figure 3), suggesting that the 141R and 153G variations in the NH_2_-terminal region do not affect the amine oxidase activity of LOXL1.

## Discussion

Since the first report on genetic association of rs1048661 and rs3825942 with XFG in Nordic populations [13], reversal of the risk alleles have been reported in various ethnic backgrounds. For instance, the G allele of rs1048661 was identified as a risk allele in most populations studied, including Nordic, United States, German, Italian, Caucasian Australian, Finnish, and black South African populations [13-17,20], while the opposite T allele of rs1048661 was reported to increase the risk of XFG in Japanese and Chinese populations [21,22]. The G allele of rs3825942 has been reported as a risk allele in most ethnic backgrounds studied [13-17,21-26]; however, the A allele of rs3825942 was identified as a risk allele for XFG in black South Africans [20]. In addition, an association between rs1048661 and XFG was not observed in some populations, including Indian, Chinese, Latin American, and United States populations [23-26]. These inconsistent genetic findings suggest that the R141L and G153D variations may not be causative factors in the development of XFG. To investigate the effects of the R141L and G153D variations on amine oxidase activity of LOXL1, we generated four different haplotype variant proteins with R141L and G153D. In amine oxidase assays using the purified LOXL1 variant proteins, the four different haplotypes did not show significantly different amine oxidase activities toward elastin, type I collagen, or cadaverine. These assay results explain the discrepant genetic findings on the association of rs1048661 and rs3825942 with XFG in different ethnic backgrounds, suggesting that other unknown genetic factors or molecular mechanisms may be more critical in the development of XFG.

Compared to the highly conserved COOH-terminal domains of the LOX family proteins, LOXL1 shows little amino acid sequence homology with LOX in the NH_2_-terminal region. Both LOX and LOXL1 are secreted into the extracellular matrix and become enzymatically active after proteolytic cleavage of the NH_2_-terminal propeptide region by BMP-1 [19,27]. Using a recombinant protein approach, the NH_2_-terminal propeptide region (codons 1 to 303) of LOXL1 was shown to play a critical role in interaction with tropoelastin, directing the LOXL1 protein onto elastic fibers [28]. Thus, the presence of the R141L and G153D variations in the propeptide region raises a possibility that these missense variations may be important in directing LOXL1 onto the sites of elastogenesis by influencing its interaction with tropoelastin. Alternatively, the missense variations of R141L and G153D may affect the interaction of LOXL1 with the scaffolding proteins required for elastogenesis. For instance, fibulin-5, a microfibril-associated protein, is critical for elastic fiber organization, functioning as a scaffold element for elastogenesis [29,30]. Further, fibulin-5 colocalizes and interacts with LOXL1, through the NH_2_-terminal region unique to LOXL1 [8]. These findings suggest that the R141L and G153D variations may affect the interaction of LOXL1 with fibulin-5, thus leading to disorganized formation and abnormal aggregation of elastic fibers. Further molecular studies on the effects of R141L and G153D on proteolytic processing of LOXL1 or interactions with tropoelastin and the microfibril-associated proteins will be necessary for resolving these intriguing questions.
